# Words before pictures: the role of language in biasing visual attention

**DOI:** 10.3389/fpsyg.2024.1439397

**Published:** 2024-12-18

**Authors:** Giulia Calignano, Anna Lorenzoni, Giulia Semeraro, Eduardo Navarrete

**Affiliations:** Department of Developmental Psychology and Socialization, University of Padova, Padua, Italy

**Keywords:** linguistic labels, target detection, spatial cueing, eye-tracking online, generalized additive mixed model, novel objects, pseudoword, Posner cueing paradigm

## Abstract

**Background:**

The present study investigated whether semantic processing of word and object primes can bias visual attention using top-down influences, even within an exogenous cueing framework. We hypothesized that real words and familiar objects would more effectively bias attentional engagement and target detection than pseudowords or pseudo-objects, as they can trigger prior knowledge to influence attention orienting and target detection.

**Methods:**

To examine this, we conducted two web-based eye-tracking experiments that ensured participants maintained central fixation on the screen during remote data collection. In Experiment 1, participants viewed a central prime—either a real word or pseudo-word—followed by a spatial cue directing them to a target on the left or right, which they located by pressing a key. Experiment 2 presented participants with real objects or pseudo-objects as primes, with primes and targets that either matched or did not match in identity. Importantly, primes in both experiments conveyed no information about target location.

**Results:**

Results from Experiment 1 indicated that real word primes were associated with faster target detection than pseudo-words. In Experiment 2, participants detected targets more quickly when primed with real objects and when prime-target identity matched. Comparisons across both experiments suggest an automatic influence of semantic knowledge on target detection and spatial attention.

**Discussion:**

These findings indicate that words can contribute to attentional capture, potentially through top-down processes, even within an exogenous cueing paradigm in which semantic processing is task-irrelevant.

## Introduction

Object detection and recognition are influenced by contextual variables. A classic example is the perception of ambiguous objects in different contexts. For instance, the Bruner and Minturn’s (1955) well-established “B/13” object is judged as “13” when embedded between “12” and “14,” but as “B” when between “A” and “C.” Such an effect has been explained by the fact that prior knowledge about an object biases the visual processing of such an object, whose identity is perceived as a number or a letter depending on the context in which it is embedded (Panichello et al., 2013). Those contextual effects are presumably supported by feedback to visual areas from higher level cortices (Bar, 2004). That is, there are widespread top-down influences on human attention and perception (Eimer, 1996, 2014) in a way that our prior representations about numbers or letters, can affect object processing (Biderman et al., 2020).

In the realm of the link between mental representation and attention, at least two attentional modalities reflect a distinction between top-down and bottom-up processing. In bottom-up processes, attention is captured by perceptually salient stimuli; in contrast, top-down processes require cognitive control to direct attention toward task-relevant information, guided by prior knowledge or expectations (Desimone and Duncan, 1995; Yantis, 2000). For instance, while observing a complex scene, bottom-up cues (e.g., a bright red object) might draw one’s attention involuntarily, whereas top-down attention is engaged if the task requires locating a specific item based on memory or instruction. Our study manipulated both top-down and bottom-up cues to examine how attention is allocated in linguistic and visual tasks.

Visual attention evidence have extensively supported that pictures effectively prime exogenous attention through distinctive perceptual cues (Spruyt et al., 2009). There is widespread consensus on the fact that visual stimuli with distinctive perceptual features capture attention depending on both their bottom-up features, but also depending on the task goals that is, depending on top-down priorities (Theeuwes, 1992; Eimer, 1996; Wolfe and Horowitz, 2004). Picture-based studies add critical insights into how visual stimuli finely drive reflexive (exogenous) and proactive (endogenous) attention, especially in contexts requiring rapid identification and processing of meaningful content (Eimer, 1996; Carrasco, 2011; Corbetta and Shulman, 2002; Henderson, 2003; Kochari, 2019; Konkle et al., 2010; Petersen et al., 1989; Posner, 1980; Posner and Cohen, 1984; Posner and Petersen, 1990; Posner et al., 1985; Posner et al., 1989).

Furthermore, several studies have indicated the existence of attentive guidance processes primed by words (Hutchison et al., 2013). Since infancy, we make strategic use of words to organize events in space even in the absence of any visual referent (Ganea et al., 2007; Saylor, 2004; Osina et al., 2018; Willits et al., 2013; Vales and Smith, 2015). In a recent event-related potential (ERP) study with 10- to 12-month-old infants, Cosper et al. (2020) presented infants with environmental sounds and pseudo-words in both consistent and inconsistent pairings. The results show word-form familiarity effects, manifested as consistent pairings effects in ERPs, precisely timed with the initiation of the pseudo-word. Also in adults there is extensive literature suggesting that words prime conceptual representations that efficiently guide object detection and recognition (Maxfield, 1997). For instance, in a simple visual detection task, Lupyan and Spivey (2010a) asked participants to detect masked target letters (e.g., M). The letter could be preceded by an auditory cue containing the name of the letter (“emm”) or by silence. The results showed an increased visual sensitivity predicted by the letter naming condition compared to silence. In the same vein, Lupyan and Ward (2013) indicated that hearing a verbal label (‘zebra’) helps participant become aware of objects that were presented in a visual degraded condition (i.e., continuous flash suppression) [see, for further evidence and discussion, Lupyan et al. (2020), Calignano et al. (2021), and Kan and Thompson-Schill (2004)].

Of notice, among several paradigms, the Visual Word paradigm has provided a solid framework for examining how attention is modulated by linguistic stimuli (Rayner, 1998). The epistemic rationale of the Visual Word paradigm expect that when participants hear spoken language alongside a visual display of objects, they tend to fixate on the visual referents associated with the words, driven by semantic overlap. In a classical experiment with the Visual Word paradigm, participants heard the word “piano” and fixated on a piano among unrelated distractors, a trumpet among distractors, or both a piano and a trumpet, with fixations increasing as the word unfolded. These kind of studies concludes that eye movements are influenced by the degree of match between a word and the mental representations of objects, going beyond just visual form (Huettig and Altmann, 2005). Also, Salverda and Altmann (2011) reported that in visual detection and discrimination tasks, the presentation of spoken words denoting a distractor captures attention by guiding visual attention versus the referred stimulus. These authors suggested that words automatically guide visual attention toward a visual scene even when they are task irrelevant. In a more recent study by Rogers et al. (2017a), adult participants were exposed to two novel objects during a familiarization phase, with one object labeled with a novel term (e.g., zeg) and the other object unlabeled. Immediately following this phase, participants performed a modified version of the Posner cueing task (Posner, 2016), where target locations were spatially pre-cued with either the labeled or unlabeled objects. The target location could either match the pre-cued object or differ from it. Results indicated faster target detection times when targets were cued with labeled objects compared to unlabeled objects, suggesting that labeled objects trigger an attentional capture effect. However, those findings did not indicate that words can capture and prioritize spatial locations when the goal of the task is to locate a pre-cued target, and when words do not provide any spatial information about the target location.

In the present study, by contrasting words and images, we focus on the modality-specificity of semantic processing in attentional guidance, providing insights into the generalizability of semantic effects across different representational formats. Our study utilizes Posner’s attentional cueing paradigm (see Posner, 2016) to explore both exogenous and endogenous attention. In this framework, exogenous attention is engaged by the sudden appearance of peripheral spatial cues that capture participants’ attention reflexively, and that is the only relevant cue for the task. For endogenous attention, participants were given a prime that has no relevance in the task but that they could encode and interpret, directing attention toward the prime identity (Lucas, 2000; Maxfield, 1997). Nevertheless, the prime was completely irrelevant to perform the task. This study aimed to understand how semantic prime, whether conveyed through words or pictures, biased visual attention within an exogenous cueing paradigm in which participants had to locate a target on the right or on the left of a central fixation point. Experiment 1 investigates the influence of written words versus pseudo-words on attention, while Experiment 2 examines if similar effects occur with object primes.

Specifically, in Experiment 1, participants were instructed to identify the location (left or right) of a target object stimulus. A prime word or pseudo-word was presented at the screen’s center before the spatial cue. Given that written words typically elicits automatic semantic processing (Kinoshita and Lupker, 2004; Neely, 1977). Our goal was to examine whether semantic processing influences overall target detection time. To achieve this, we compared response times between trials where semantic information was provided (word prime stimulus) and a control condition where no semantic information was provided (pseudo-word prime stimulus). Experiment 1 aimed to explore whether non-relevant words lacking spatial information could still guide target detection. We hypothesized a main effect of word type (words vs. pseudowords), reflecting the influence of semantic processing on target detection. Specifically, we expected words to facilitate target detection more effectively than pseudowords. Additionally, we anticipated an interaction between cue validity and prime type (words vs. pseudowords). This interaction would indicate the differential impact of words and pseudowords on spatial orienting, with words potentially enhancing orienting effects in valid cue conditions more than pseudowords.

Notably, our study represents the first investigation of this phenomenon within a spatial exogenous cueing task (Posner task). A critical aspect of our empirical approach is the type of words we selected as prime stimuli. It is well-documented in the literature that words denoting spatial information (e.g., left, right, up), as well as words denoting referents associated with specific locations (e.g., shoe-down, sun-up), guide visual attention (Meier and Robinson, 2004; Pecher et al., 2010; Šetić and Domijan, 2007; but see also Petrova et al., 2018). Since we were interested here in the influence of words *per se* (i.e., as linguistic labels) in guiding attention, we chose prime words without spatial connotation. It is worth noting that words without spatial semantic features may not affect spatial selection priority during an exogenous task (Theeuwes, 2010). Here, we aimed at challenging this assumption by investigating whether the presentation of a written word biases target detection and spatial selection priority when word meaning is irrelevant, and its location does not prime the target’s location. Indeed, a second critical aspect of our research is the choice of an exogenous cueing paradigm. That is, before the target was presented, a spatial cue (X) briefly appeared either at the eventual location of the target (valid trials) or at a specific alternate location (invalid trials). Exogenous cueing tasks are expected to preferentially guide visual attention via stimulus-driven processes, that is, by a bottom-up capture (Mulckhuyse and Theeuwes, 2010; Theeuwes, 2013). Therefore, the choice of a task mainly based on the bottom-up capture of attention reinforces the notion that we are investigating the influence of word-label during target detection and spatial orienting.

Accordingly, we were interested in capturing the temporal dynamics of the effect. To provide such a temporal description, we selected a statistical approach for response time particularly apt to model the likely nonlinear relationship between response time variation and the time course of the experiment. This is, Generalized Additive Mixed-effects models (Baayen and Linke, 2021; Baayen et al., 2017). Two experiments were presented. Data were collected remotely, and additional online eye-tracking data were used as a methodological attention check. Of note in this study, eye-tracking data served as a crucial validity check by helping us identify and exclude participant trials where central fixation was not maintained, thus enhancing the overall reliability of the measurements of Valid and Invalid trials.

The research protocol was approved by the Ethics Committee of the Department of Developmental Psychology and Socialization, University of Padova (protocol number: 3819). All stimuli, data and the analysis pipeline are openly available at the repository link (https://osf.io/qt3bf/?view_only=f6069439a3884985b6920b7d74db34a4).

## Experiment 1

### Method

#### Participants

50 adults (27 women, mean age in years = 27.85, SD = 7.19) Italian participants were recruited through the Prolific crowdsourcing platform (Palan and Schitter, 2018). We excluded six participants who showed more than 80% missing data (see below the Eye-tracking attention check section). The final sample included 46 adults (23 women, mean age in years = 30.38, SD = 6.12). Participants accessed the experiment remotely by clicking on a provided link, ensuring participation was fully online. The inclusion criteria for all participants were to be in good health and to have no sensory or neurological disorders. Notably, to ensure that statistical results are representative of the population to which they are assumed to generalize, it is good practice to conduct *a priori* power analysis. However, the magnitude of spatial orienting measured through the Posner paradigm has been found to vary with increased trial sampling (e.g., Weaver et al., 1998; Pratt and McAuliffe, 1999; Lupiáñez et al., 2001), reducing the usefulness of any power analysis *a priori.* In addition, the average effect of exogenous cue over time in the entire experiment has not been synthesized in previous studies, neither the impact of prime words presented prior to an exogenous spatial cue in a remote eye-tracking detection task. Given these difficulties, *a priori* power analysis was not allowed in this case, and we adopted a different strategy. For regression analyses, it has been proposed that increasing 5–10 observations per variable is likely to provide an acceptable estimation of regression coefficients, standard errors, and confidence intervals (Bates et al., 1987; Bollen, 1989; Hanley, 2016; Knofczynski and Mundfrom, 2008). We followed this suggestion and fixed the sample size at 50 participants since our experiment involved 5 variables, i.e., Cue, Prime, participant, stimuli and trial number, in a repeated measure design.

#### Apparatus

To measure the visual behavior of the participants at home in a controlled experimental task, we used open-source software based on deep learning modeling for webcam-based eye-tracking (Finger et al., 2017; see https://github.com/Labvanced). The software has shown good accuracy and realistic precision compared to other online experiment platforms (e.g., Anwyl-Irvine et al., 2021) and has been successfully used to collect at-home adult eye-tracking data (Kaduk et al., 2023).

### Materials

#### Prime stimuli

Prime stimuli were composed of 20 words and 20 pseudo-words. Twenty Italian words depicting concrete objects were chosen as experimental prime stimuli. Lexical frequency and letter length, two psycholinguistic variables that have been shown to affect word recognition, were extracted from the PhonItalian database (Goslin et al., 2014). Words had a mean lexical frequency of 21.5 (SD = 82.24) and a mean letter length of 7.5 (SD = 2.09). The 20 pseudo-words were created by using words of similar frequency (mean = 75.95, SD = 93.07) and letter length (mean = 7.5; SD = 1.29) of the 20 prime words. The pseudo-words were created by altering one of the letters of the words. The position of the letter change (beginning, middle, or end of the word) was counterbalanced across pseudo-words.

#### Target stimuli

Target stimuli were composed by 20 objects from the Massive Memory Unique Object Images (Brady et al., 2008) and 20 novel objects from the Novel Object and Unusual Name (NOUN) Database (Horst and Hout, 2016). For the pool of objects, two different photographs of the same object (i.e., two exemplars) were selected. Similarly, the same was done for the pool of novel objects. In total, target pool was composed by 40 photographs of objects and 40 photographs of novel objects. The two exemplars of a target object were presented with the corresponding prime word, while the two exemplars of a novel object were presented with the same pseudo-word. Novel objects were randomly assigned to a pseudo-word.

### Procedure

**Figure 1:**
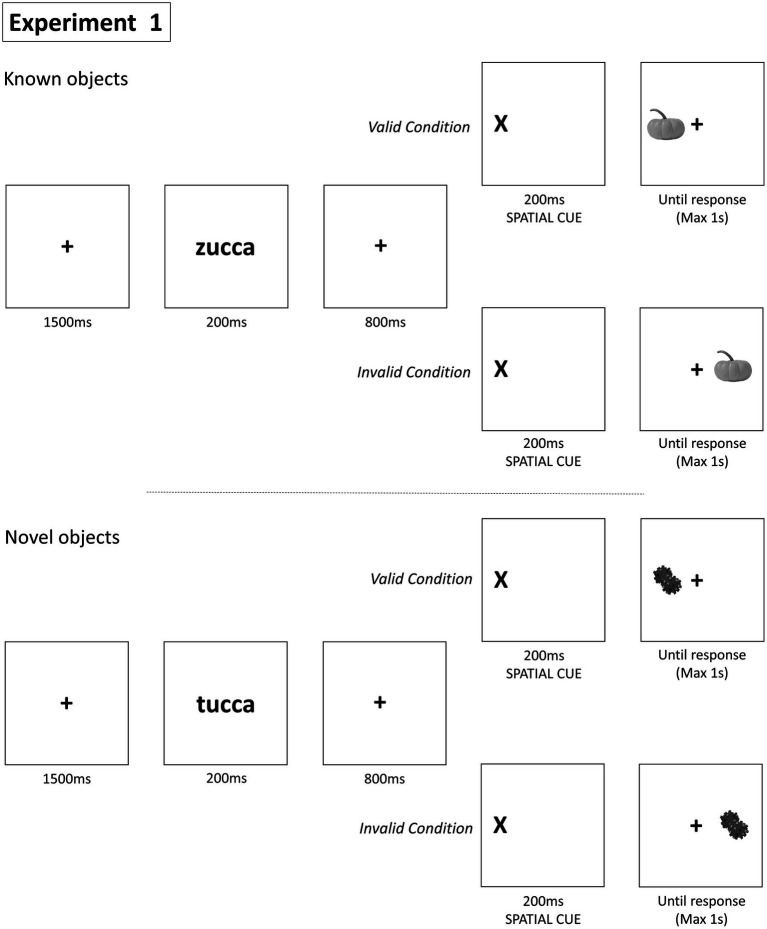
Paradigm of Experiment 1. This figure illustrates the procedure of the attention task using known and novel objects. For known objects, the sequence involves a fixation cross, a word cue (‘zucca’), a spatial cue, and the target object. In the valid condition, the object appears in the same location as the cue. In the invalid condition, it appears in a non-indicated location. For novel objects, the sequence is similar but uses unfamiliar and unrelated terms and images (‘tucca’). The task measures the ability to shift attention based on spatial cues and the familiarity of the object, assessing response times and accuracy within a 1-s response window.

After reading the instructions, the participants engaged in the calibration procedure (130 points, 9 poses, around 5.5. minutes). They ensured that their devices had a minimum screen resolution of 600 × 600 pixels and were running on Windows, Mac, or Linux. The procedure involved fixating on 15 dots displayed on the screen for approximately 15 s, while their head pose was being calibrated. The computer’s face processing capability of 7.5 Hz determined the speed of gaze sampling, with higher sampling modes being faster. The recommended setting was medium, a trade-off between precision performance and participant accessibility. The study had a 2 Cue (Valid vs. Invalid) × 2 Prime (Words vs. Pseudo-words) within repeated measures design. Before starting the actual experimental session, participants pass through a training phase to familiarize participants with the Posner task. Figure 1 shows that each trial began with the presentation of a black fixation cross at the center of the screen lasting for 1,500 ms, followed by a central picture of an object for 200 ms. Right after the fixation cross appeared for 800 ms, the spatial cue was presented on the left or the right to the center of the screen for 200 ms. The inclusion of a second fixation cross, displayed for 800 ms before the spatial cue, serves to reorient and stabilize participants’ gaze, ensuring consistent initial conditions across trials. This helps in minimizing the influence of previous visual stimuli, allowing for more accurate measurement of participants’ responses to the spatial cue. Lastly, the target stimulus appeared in one of two possible locations until a response was provided or for a maximum of 1,000 ms. The spatial cue was Valid when presented in the same location as the target and Invalid when in a different location. Participants were instructed to press a key of a keyboard if the target was on the left and a different key if the target was on the right. Participants had the option to take a break as the experiment would pause if they moved away from the screen; otherwise, it continued uninterrupted for 15 min. The response keys used in the experiment were ‘Z’ and ‘M’. There were 36 trials for each level of the 2 × 2 factorial design, resulting in a total of 144 trials presented in random order (see Figure 1).

### Statistical analysis

We used Colab, an on-line platform that allows us to use the Python 3.6 language on the chosen browser (Bisong, 2019), and the R software (R Core Team, 2020) as tools for data management and statistical analysis, respectively. We excluded anticipatory responses (<100 ms) and included response times up to 1,000 ms to remove outliers and potential artifacts unrelated to the cognitive process under investigation. This approach ensures the quality and reliability of our data by focusing on responses within a reasonable time frame to be associated with detection mechanisms (Ratcliff, 1993). Error trials (2%) were excluded from the RTs analysis. We analyzed an average of 135.39 trials (SD = 12.63) per participant. Data were analyzed using generalized additive mixed effects models (GAMMs) for response time and generalized linear mixed effects models (GLMMs) for response accuracy, with the mgcv package (Wood and Wood, 2015, version 1.8–38;) and the lme4 package (Bates et al., 2015, version 1–27.1), respectively. Since residuals are often positively skewed and heteroscedastic when dealing with nonnegative behavioral data (e.g., response time and accuracy), these models are preferred to the classical ANOVAs and general linear models (GLMs).

To find the best approximation to the true model, we followed a model comparison approach with AIC (Akaike Information Criterion) and AIC weight as goodness-of-fit indexes. The AIC and AIC weight compare all the models at once and give information on a model’s relative evidence (i.e., likelihood and parsimony), so that the model with the lowest differential AIC and the highest AIC weight is to be preferred (Wagenmakers and Farrell, 2004). We started from the simplest model with only random factors and proceeded by adding predictors and weighting the effects of the main manipulations. This approach ensured us we could test if adding interaction or secondary variables improves the model as detailed below for each task and dependent measures. In the provided GAMM models, there were both random and fixed factors included. The random structure models the subject-specific intercept and slope to account for individual variations in reaction time. The fixed factors were Cue (Valid vs. Invalid) and Prime (Word vs. Pseudo-word). Models also included smooth functions of trial order (i.e., Trial_Nr) to capture non-linear relationships over time. By model comparison we could assess the impact of including different fixed factors and interaction terms on the reaction time outcome, enabling a deeper understanding of the underlying relationships in the data.

Finally to explore the temporal dynamics of the influence of words in guiding visual attention, we analyzed the response time by means of GAMMs that allows us to observe if the effect is stable over time. The model is estimated using penalized regression techniques, which help to avoid overfitting and produce more reliable predictions.

### Eye-tracking attention check

We analyzed data points corresponding to participant fixations at specific areas of the screen over the time course of each trial. We did so by taking advantage of arbitrary coordinate units describing the x-axis of the screen and tracking fixations across time to check that they correspond, respectively, to (a) the central fixation cross (400 x-axis coordinate), (b) the area of the left exogenous cue (199 x-axis coordinate), and (c) the area of the right exogenous cue (599 x-axis coordinate). Supplementary Figure S1 in the supplemental materials shows that most fixations fell within the area of the fixation cross (400 x-axis coordinate) independent of the trial number and the identity of the trial over time. This fundamental check, useful for at-home study, ensures that participants performed the task as expected, maintaining central fixation, as is customary in traditional cueing tasks. Four participants who showed more than 80% of missing eye-tracking data were excluded from further analyses.

### Results

#### Response time

**Figure 2:**
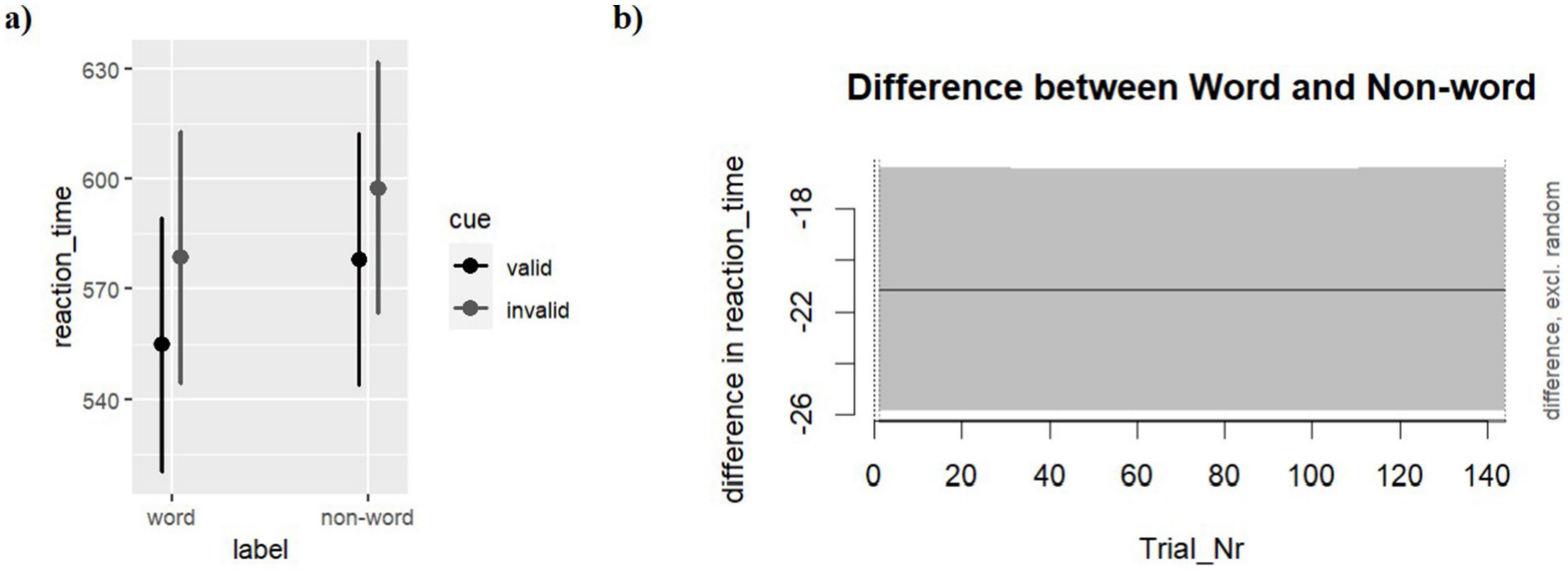
Reaction times **(A)** estimated effects and the 95% confidence intervals for cue (Valid vs. Invalid) and Prime (Word vs. Pseudo-words). **(B)** Differential effect plots showing the subtractive effect of Word minus Pseudo-words trial-by-trial. The gray area shows the 95% confidence intervals around the estimated mean effect over time. The whole-time window falling within the vertical dot lines indicates that the differences between conditions were significantly different from 0 across the whole experiment.

Table 1 shows the descriptive statistics of reaction time across the conditions of the design. The model (M1) with the fixed factor Cue and Prime emerged as the most plausible predicting response time (for model comparison see Table S1 in supplemental materials). The Valid cues predicted a shorter response time compared to the Invalid cues (*b* = −13.69, SE = 2.40, *t* = −5.702, *p* < 0.0001). The results also showed a significant and stable effect of Word predicting faster detection compared with Pseudo-words (*b* = −21.12, SE = 2.40, *t* = −8.801, *p* < 0.001) across the whole-time course of the experiment. Looking at the interactive model, no substantial interactive effect emerges (*b* = −4.36, SE = 4.78, *t* = −0.91, *p* = 0.362). Figure 2 shows the effects estimated by the best model, M1, and the associated 95% confidence intervals around the estimated mean effect over time.

**Table 1:** Average and standard deviations in brackets of reaction times in milliseconds divided by Cue and Prime conditions in Experiment 1.

Prime		
Cue	Word	Pseudo-word
Valid	445 (111)	468 (130)
Invalid	461 (119)	480 (131)

#### Accuracy

The model M1 with Cue (Valid vs. Invalid) and Prime (Word vs. Pseudo-words) emerged as the most plausible predicting accuracy. The results of the logistic regression show a significant effect of the Invalid trials predicting a reduced accuracy compared to Valid trials (*b* = − 3.43, SE =0.46, *z* = −7.50, p < 0.001), whereas no difference in accuracy emerged by comparing words and pseudo-words (*b* = 0.16, SE = 0.17, *z* = 0.94, *p* = 0.348).

## Discussion

In Experiment 1, target detection occurred more quickly in the presence of prime words compared to pseudo-words. Specifically, this facilitation found with words persisted from the initial trials and remained significant and stable throughout the entire experiment. These results suggest that words amplify the processing of subsequent exogenous cues, despite their presentation as prime stimuli being irrelevant to the task and lacking predictive information regarding target location. It is notable that words improved the effectiveness of target detection relative to pseudo-words under two critical conditions: first, when word presentation was non-informative and did not predict target location, and second, when the task focused on target detection rather than object recognition.

Nevertheless, prior studies have indicated that the efficacy of top-down information in guiding visual attention relies on its capacity to retrieve a detailed representation of the target object. For example, in visual search tasks, a cue object provides information about the target on a trial-by-trial basis. It has been observed that discrepancies in size, orientation, or shape between the cue object and the target can reduce search speed (Arita et al., 2012; Eimer, 2014; Vickery et al., 2005; Wolfe and Horowitz, 2004). In the context of our research, presenting a prime word (e.g., DOG) may activate a less precise semantic representation compared to visually presenting an exemplar of that specific object (i.e., a picture of a dog; see Wolfe et al., 2004; Eimer, 1996). To investigate the influence of semantic knowledge itself on attention capture in Experiment 1, we replicated the previous experiment and manipulated the perceptual exactness vs. semantic correspondence between prime and target elements. In Experiment 2, we introduced a Match/Mismatch condition where the prime picture could either match the exact picture of the target (the same example of a dog) or a different exemplar from the same category (two examples of dogs). Consistent with previous research (e.g., Wolfe et al., 2004), we hypothesized that the Matching condition would result in greater attention capture compared to the Mismatching condition.

Experiment 2 mirrored the Words and Pseudo-word condition of Experiment 1, but with the addition of Known and Unknown objects. Consistent with previous findings (Eimer, 1996, 2014), we anticipated that the clearer the representation of the prime target, the more accurate the target detection would be. Accordingly, we predicted that Known objects would retrieve not only perceptual but also semantic top-down representations, while Unknown objects would only trigger perceptual representations. Consequently, Known objects were expected to activate more elaborate semantic representations compared to Unknown objects, likely resulting also in enhanced spatial orienting. Experiment 2 replicated Experiment 1’s design precisely, with the sole variation being the type of prime used (word vs. picture). It is crucial to note that all target images used in both experiments remained consistent throughout the study, with only the type of prime differing.

The theoretical distinction between semantic and perceptual representations is critical to understanding how different forms of information guide attention, and specifically target detection and spatial orienting. Perceptual representation refers to sensory features, such as color, shape, or spatial location, whereas semantic representation involves meaning or category-related information. These distinctions influence spatial attention differently: perceptual cues often drive exogenous attention (e.g., focusing on the brightness of an object), whereas semantic knowledge can modulate endogenous attention, directing focus to an object based on category relevance (Peelen and Kastner, 2014). In the context of spatial attention, perceptual cues facilitate immediate, bottom-up attention shifts, while semantic knowledge guides attention in a goal-directed, top-down manner. Experiment 2 explores how these distinctions operate across modalities, specifically how pictures engage semantic knowledge differently from words. The shift from words to pictures in Experiment 2 allows us to examine whether the richer perceptual information in images enhances exogenous attentional engagement and whether the availability of semantic context in visual stimuli aids in guiding endogenous attention more effectively.

Specifically, for Experiment 2, we hypothesized that (1) real objects would facilitate faster target detection than pseudo-objects due to the presence of meaningful semantic information, (2) perceptual congruency between primes and targets would enhance attentional engagement, and (3) an interaction would occur where matched real objects would yield the fastest detection times, reflecting an optimal combination of semantic and perceptual alignment.

## Experiment 2

### Method

#### Participants

50 adults (39 females, mean age in years = 27.29, SD = 9.37) were recruited. Seven participants were excluded because they showed poor eye-tracking data (see Statistical Analysis). Our final sample comprised 43 participants (33 women, mean age in years = 27.26, SD = 9.29).

#### Apparatus

Identical to Experiment 1.

#### Materials

The known and unknown objects were identical to Experiment 1.

### Procedure and statistical analysis

**Figure 3:**
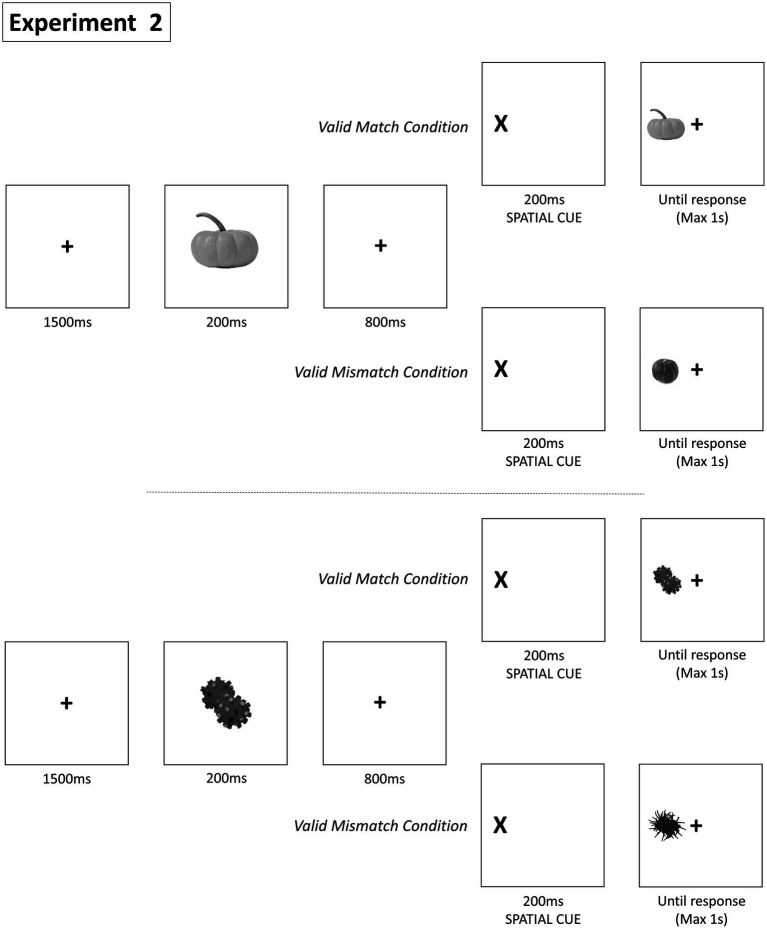
Paradigm of Experiment 2. Each trial presents a fixation point, an object, a spatial cue, and a final object for participant response. Conditions vary based on object match and spatial cue validity, with ‘Valid Match’, ‘Invalid Match’, and ‘Valid Mismatch’ and “Invalid Mismatch” scenarios. Invalid conditions are not presented.

The same procedure as in Experiment 1 was applied here with the following differences. Experiment 2 had a 2 Cue (Valid vs. Invalid) × 2 Target-Match (Match vs. Mismatch) × 2 Prime (Known vs. Novel) within repeated measures design. In Match trials each picture presented before the spatial cue was identical to the target, and in Mismatch trials it was a different exemplar of the same object. Finally, in Know trials the presented picture corresponded to a Known (real) object while in Novel trials to an unreal object (see Figure 3). The same statistical analyses were performed as Experiment 1.

### Eye-tracking attention check

Like in Experiment 1, most fixations fell in the area with the central fixation cross (see Figure S2 in supplementary materials). We excluded seven participants who showed more than 80% missing eye-tracking data.

## Results

### Response time

**Figure 4:**
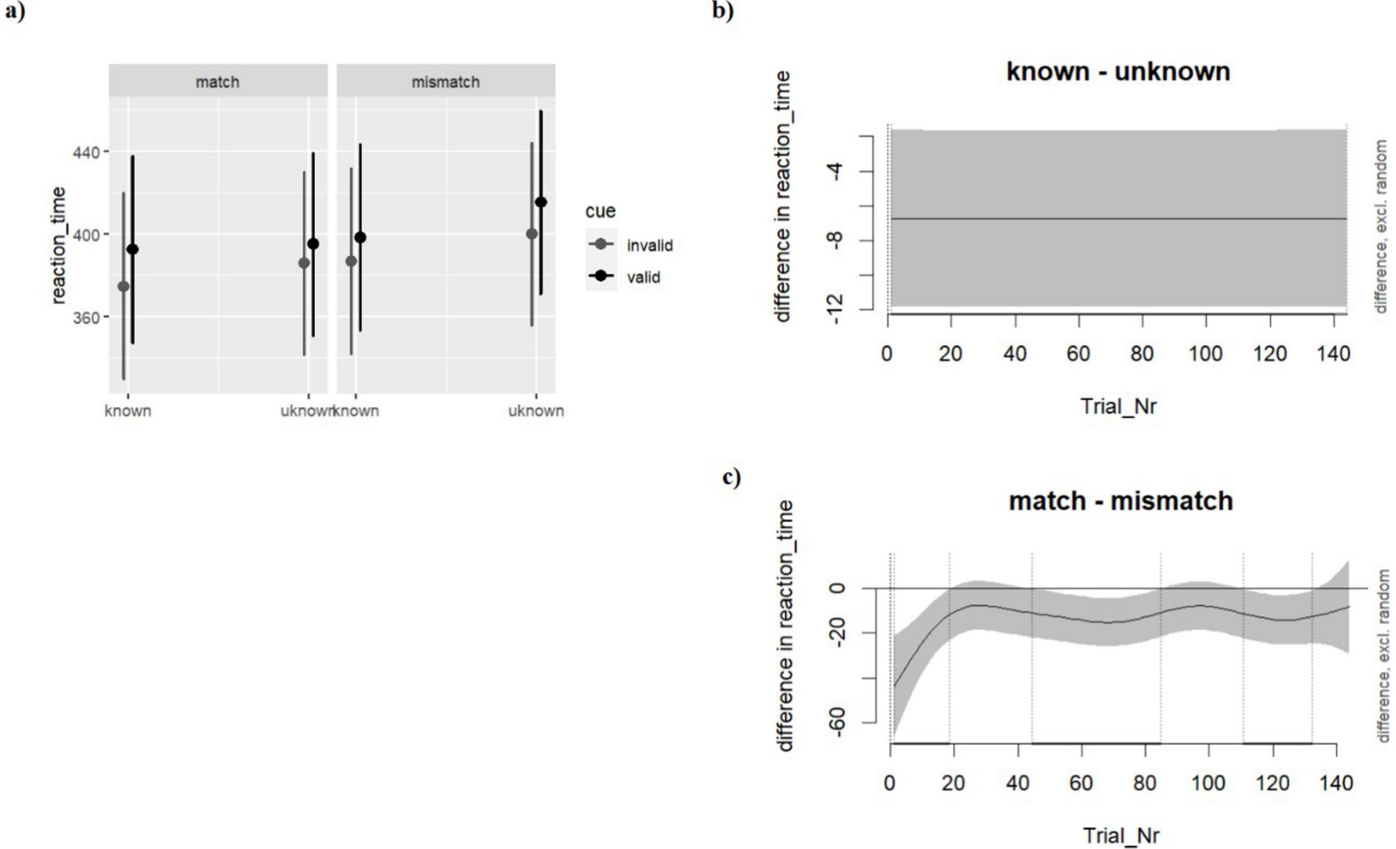
Experiment 2, response times. **(A)** estimated effects with the 95% confidence intervals of Cue (Valid vs Invalid), Prime (word *vs* pseudoword), and Match (Match vs Mismatch); Differential effect plots showing the subtractive effect of **(B)** Known-Unknown and **(C)** Matching-Mismatching condition. The gray area shows the 95% confidence intervals around the estimated mean effect over time. The time windows falling within the vertical dotted lines indicates the differences in which conditions were significantly different from 0 across the whole experiment.

Table 2 shows the descriptive statistics of reaction time across the levels of the design. As shown in Figure 4, the additive model, M1, with Cue (Valid vs. Invalid), Prime (Known vs. Unknown objects) and Match (Match vs. Mismatch) as fixed factors emerged as the most plausible model predicting response time (see Supplementary Table S2 in supplemental materials). The results show a significant effect of Cue with the Valid cue predicting longer reaction times compared to the Invalid trials (*b* = 7.23, SE = 2.59, *t* = 2.788, *p* < 0.001). Furthermore, we found a significant effect of Known objects predicting a faster target detection compared to Unknown objects (*b* = −6.72, SE = 2.59, *t* = − 2.60, *p* = 0.009). In addition, we found a substantial effect of the Matching trials predicting faster response time compared to Mismatching trials (*b* = −12.88, SE = 2.60, *t* = −4.944, p < 0.001). Of note, such an effect emerges since the first trials and throughout the experiment. Finally, by looking at the interactive model, no substantial interactive effect emerged.

**Table 2:** Average and standard deviations in brackets of reaction times in milliseconds divided by Cue, Prime, and Match conditions in Experiment 2.

Prime				
Cue	Known		Unknown object	
	Match	Mismatch	Match	Mismatch
Valid	480 (136)	485 (138)	480 (133)	498 (142)
Invalid	469 (141)	485 (143)	478 (142)	485 (145)

### Accuracy

The model M1, with Cue (Valid vs. Invalid), Prime (Known *vs* Novel object) emerged as the most plausible model predicting accuracy. The results showed a significant effect of the Invalid trials predicting a reduced accuracy compared to the Valid trials (*b* = −1.12, SE =0.17, *z* = −6.42, p < 0.001). No substantial difference emerged by comparing Known to Unknown objects (*b* = − 0.19, SE = 0.16, *z* = −1.24, *p* = 0.214) and Matching to Mismatching trials (*b* = −0.06, SE = 0.16, *z* = − 0.41, *p* = 0.691)^1^.

## Discussion

The results of Experiment 2 showed that the Known objects predicted faster responses compared to the Unknown objects. We also reported a significant effect of Match, with matching trials predicting faster responses compared to Mismatch trials. Both effects emerged early and remained stable across the whole experiment. These results suggest that top-down information in the form of semantic knowledge (Known/Unknown) and perceptual (Match/Mismatch) impacts the subsequent target detection during a spatial cueing task. It is important to note here that the Matching effect emerged independently from stimulus type, that is, it occurs when Known or Unknown objects were presented as prime stimuli. This indicate that attentional guidance can be influenced by semantic associations irrespective of modality.

Experiment 2 was designed to test the generalizability of the semantic attentional effects observed in Experiment 1 by using object primes (real vs. pseudo-objects) rather than words. The cue condition in Experiment 2 is of particular interest because it allowed us to assess attentional shifts independently of the semantic priming effects, serving as a baseline to isolate attentional mechanisms. By including this condition, we can observe that may the attentional capture observed with real and pseudo-object primes can be genuinely a perceptual effect and a result of semantic priming.

### Joined analysis experiments 1 and 2

In this section, we show additional analyses to scrutinize the patterns of reaction times observed across the two experiments. It is worth noting that the target objects in both the Words and Known objects trials, as well as the target objects in the Pseudo-words and Unknown objects trials, remained identical across experiments. Thus, the sole discrepancy between Experiment 1 and Experiment 2 lay in the format of the prime stimuli (words vs. pictures) and in the between comparison between the two tasks. To facilitate a comparison between the two experiments, we employed logistic generalized mixed-effect modeling, which effectively accounted for within-participant and between-effect variances (Cue and Prime). Interestingly, our analysis revealed no association between response time and the two distinct experiments (*b* = 0.0004, SE = 0.02, *z* = 0.021, *p* = 0.983). We proceeded to compare the manipulation effects across both experiments.

**Figure 5:**
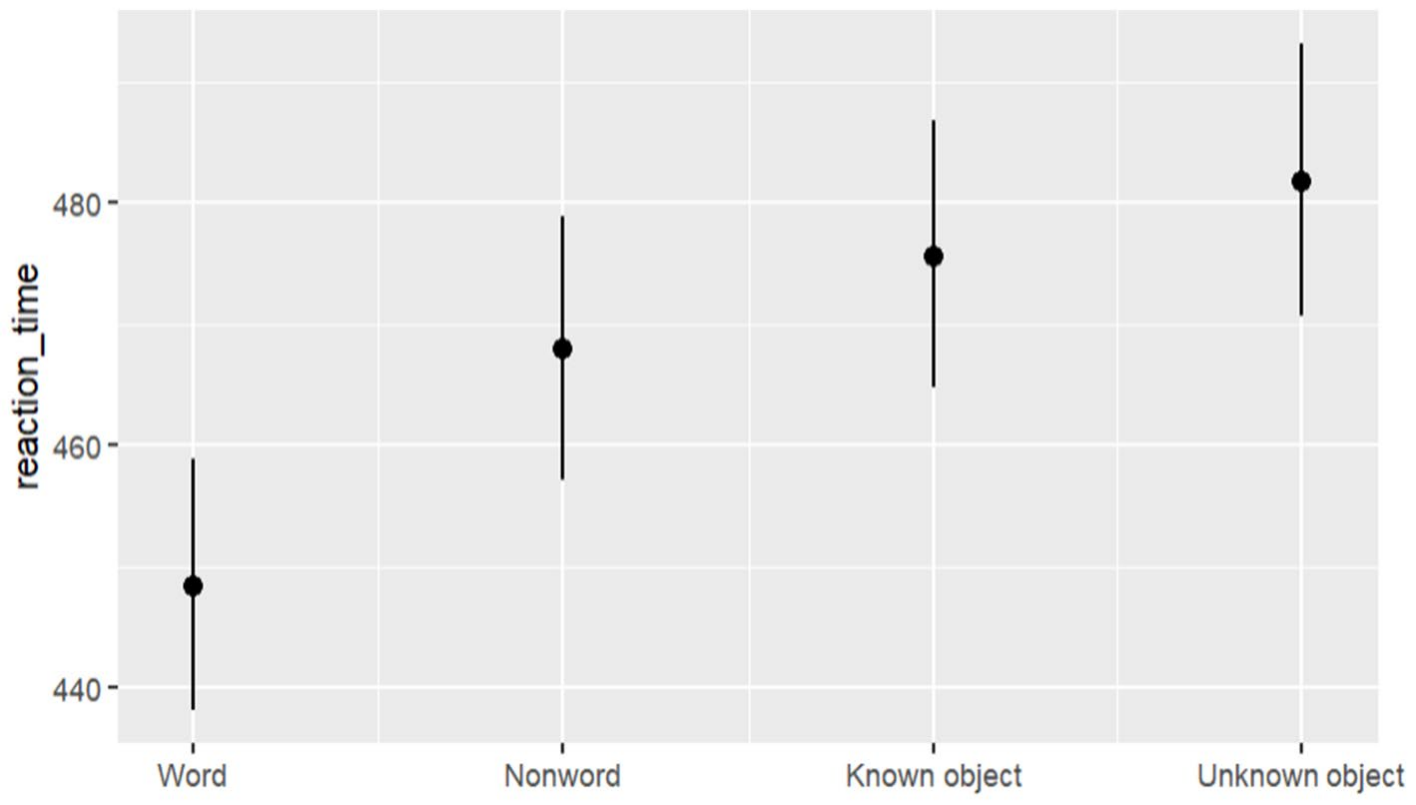
Estimated effects and 95% CI across Experiment 1 and 2 for Word vs Pseudo-words vs Known objects vs Unknown objects on reaction times in milliseconds.

The best model (M1), featuring Prime (Words, Pseudo-words, Known objects, and Unknown objects) as a fixed factor, emerged as the best predictor of response time (refer to model selection in Table S3 in supplemental materials). Our results indicated the substantial effect of Words compared to Pseudo-words (*b* = 0.043, SE = 0.007, *t* = 6.153, *p* < 0.001), Known objects (*b* = 0.059, SE = 0.016, *t* = 3.634, *p* < 0.001), and Unknown objects (*b* = 0.072, SE = 0.0167, *t* = 4.280, *p* < 0.001), as depicted in Figure 5.

In Experiment 1, words were associated with reduced target detection time across stimuli. Conversely, in Experiment 2, prime pictures may have been constrained by the requirement for exact match between prime and target, potentially leading to slower response times across stimuli (a mismatching cost). Through direct comparison of the impact of prime words versus prime pictures (see Figure 5), a robust word effect emerged, indicating faster reaction times with prime words compared to all other prime conditions. Also, faster reaction times following pseudo-word primes compared to real object pictures might reflect reduced processing demands. Pseudo-words, being lacking of semantic content, require less cognitive effort than real object pictures, which carry both perceptual and semantic information, potentially increasing processing load. Thus, detecting the same object in a linguistic versus perceptual priming context may impact the efficiency of spatial orienting.

## General discussion

How does language influence attention? Numerous studies have indicated that words interact with object perception and attention in both adults (Salverda and Altmann, 2011; Lupyan and Spivey, 2010a, 2010b) and children (Vales and Smith, 2015). The present study aimed to investigate the specific impact of semantic information in an exogenous cueing task. We explored whether real words and familiar objects could act as effective cues, biasing the reliability of spatial cues in capturing attention, as compared to pseudo-words and unfamiliar objects. Our investigation focused on whether language modulates spatial and target capture under conditions where words and objects are displayed centrally and do not predict target location. The results confirm this influence, highlighting a unique effect of words on attentional capture. Crucially, this effect operates independently of exogenous cueing, as shown by the lack of interaction across both studies.

Of note, in both experiments, a key methodological aspect was that participants were not required to strategically use the prime to locate the target, and to continue to look at the center of the screen (Posner, 2016). In Experiment 1, we found evidence that words (i.e., object names) were associated with enhanced target detection, even when the word was shown before the spatial cue, conveyed no spatial information, and did not predict the target’s location on the screen. To further investigate the role of words in target detection, we replicated the experiment using familiar and unfamiliar images instead of words in Experiment 2. This allowed us to distinguish the impact of perceptual priming (in matching trials) from that of semantic priming (in mismatching trials). Experiment 2 results showed that trials with familiar objects and matching primes led to faster reaction times compared to unfamiliar objects and mismatched trials. Importantly, these main effects did not interact, suggesting that semantic information alone may guide attention depending on the trial context.

One first plausible explanation for the facilitation effect observed with prime words relies on the possibility that words possess inherent attentional priority over non-linguistic stimuli due to their semantic richness and cognitive salience. Of note, such possibility of the prioritization of words over other stimuli emerges from the intersection of various theoretical frameworks in cognitive psychology and psycholinguistics, rather than being attributed to a single theorist (Nedergaard et al., 2023; Grainger and Holcomb, 2009). Moreover, such hypothesis aligns with a number of research indicating the unique role of linguistic stimuli on attentional capture (Salverda and Altmann, 2011; Lupyan and Spivey, 2010b).

The present study extends previous research by Salverda and Altmann (2011) and Rogers et al. (2017a) in several key ways. First, by employing a web-based eye-tracking method, we explored ecological validity and enabled data collection across a broader participant pool, increasing the generalizability of findings on attentional engagement with linguistic and visual stimuli. Second, our design directly contrasts linguistic (words) and non-linguistic (objects) primes, revealing that attentional capture is modulated by semantic content across modalities. Third, we explore the influence of semantic congruency between primes and targets, finding that matching primes and targets facilitate attentional engagement, which was not examined in prior work. Also, our results highlight the contribution of top-down mechanisms, showing that words are associated with enhanced attentional capture even in an exogenous task context. In fact, in Experiment 1, the main effect of Prime (words vs. pseudo-words) suggests that words facilitate target detection through semantic activation, consistent with semantic priming effects rather than bottom-up attentional guidance alone. Notably, the absence of an interaction between Prime and Cue validity supports this interpretation, indicating that words enhance attentional engagement through semantic rather than purely reflexive processes. Based on those kinds of empirical indication, Waxman and Gelman (2009) proposed that linguistic labels (i.e., words) are cues to meaning that enrich the representation of objects and entail reference rather than mere association among perceptual features. Such linguistic representations are expected to guide visual attention from infancy (e, g., Fernald et al., 2008) into adulthood (e.g., Tanenhaus et al., 1995).

However, it can be argued that the present study do not provide sufficient evidence for a causal link between word processing and spatial orientation in object detection. Prime words, as opposed to pseudo-words, appear to improve target detection efficiency but not spatial selection, this is likely due to the cognitive benefits related to meaningful word processing (Sulpizio et al., 2021; Shechter and Share, 2021). The present experiment’s results—with faster response times for word and object primes and valid cues—reflect known cognitive psychology effects, suggesting separate cognitive processes rather than interaction (a word-processing effect) and quicker responses for spatial matches over mismatches (a spatial orientation effect). Although word and spatial tasks may temporally overlap, potentially causing cognitive load interactions, the lack of significant interactive effects might indicate independent functioning, that is target detection and spatial selection, not a combined causal mechanism. Of note, accuracy results show significant effects for Cue validity, suggesting that spatial relevance (validity of cues) directly improves task performance, while the type of prime (word, object) primarily influences response times rather than accuracy.

An alternative not exclusive explanation lies in the activation of semantic networks triggered by prime words. Words, as linguistic labels, not only represent individual objects but also activate rich semantic networks associated with those objects. In Experiment 1, the presentation of prime words and in Experiment 2, the presentation of known objects, may have facilitated faster target detection by priming semantic representations related to the denoted objects. This semantic activation could enhance the attentional processes by providing a cognitive context for improving target detection, also in a exogenous spatial task that does not require target recognition. This interpretation emphasizes the role of semantic knowledge in guiding attentional capture and suggests that words may serve as potent facilitators of semantic priming effects. In addition lack of interaction between the type of objects (known, unknown) and the match between the prime and target (match, mismatch) in Experiment 2 may suggest the involvement of parallel processing pathways in attentional capture. While known objects may primarily engage semantic processing pathways, leading to faster detection times, unknown objects may rely more heavily on perceptual processing pathways (Calignano et al., 2021). This dual processing hypothesis implies that the facilitation effect observed with prime words in Experiment 1 may stem from the activation of both semantic and perceptual processing pathways, resulting in enhanced attentional capture. This interpretation highlights the complexity of attentional processes and suggests that multiple processing pathways may contribute to the observed effects. Notably, the main effects of Known/Unknown and Match/Mismatch indicate that both semantic and perceptual information facilitate faster responses independently. The absence of an interaction between these factors suggests that these forms of information do not jointly modulate attentional shifts, but rather independently enhance attention. It is important at this point to remember to the reader that we expected main effects of Prime, Known/Unknown, and Match/Mismatch, reflecting faster responses when semantic and perceptual information is available. We did also expected interactions between these factors, but we did not find it as semantic and perceptual information are anticipated and likely independently enhanced attentional engagement.

Importantly, although the primes were semantically relevant, they were spatially uninformative, ensuring that any attentional effects were driven by non-predictive semantic processing rather than traditional priming mechanisms, in an exogenous cueing task. This approach differentiates our study from typical linguistic priming tasks and focuses on semantic attentional capture that does not rely on anticipatory cueing. The main effect of Match/Mismatch is likely driven by both semantic and perceptual congruency, suggesting that attentional engagement benefits from the availability of either form of congruent information. This finding highlights that both perceptual features and semantic knowledge independently contribute to enhancing attentional capture. It seems that the offered findings challenge the notion that spatial detection tasks mainly rely on bottom-up mechanisms for performance (Theeuwes, 2013). Moreover, our findings have relevant implications extending previous research findings outside the laboratory and with web-based eye-tracking that allowed for fine-graded sanity and quality check of the data. The cross-experimental finding that semantically meaningful primes (words and objects) facilitate attentional engagement supports our conclusion that semantic knowledge substantially biases the orienting system. However, real words, compared to pictures, may engage this system more effectively, likely due to differences in processing demands across formats. Our study is the first to use a detection task in which target identification is not required, and in which the prime words do not predict the target’s location. This makes the present study fundamentally different from previous studies (e.g., Rogers et al., 2017b; Stolz, 1996). A secondary insight of our research was to offered by the modeling of the temporal dynamics of word influence on visual attention using GAMMs analysis (Wood and Wood, 2015). This statistical approach allows for the visualization of effects over time, facilitating functional interpretation of repeated measures data. Such methodology has provided additional indication of the consistent, uninterrupted and distinguishable impact of word presentation within a cue-dependent exogenous orienting task.

In conclusion, our study offer a reproducible and robust experimental contexts to disentangle the distinct role of words in attentional orienting involved in object detection. We observed faster response times during target detection driven by words in exogenous cueing tasks, where neither target recognition nor discrimination is necessary. Semantic knowledge extracted from prime words guides attention, biasing rapid target detection. Overall, language can prioritize target detection independently of other perceptual information.

## Data Availability

The datasets presented in this study can be found in online repositories. The names of the repository/repositories and accession number(s) can be found in the article/supplementary material.

## Footnotes

^1^We found a sort of “validity-based inhibition of return” effect that might rely on the presentation of high-quality and detailed images in a short time window and in sequence throughout the experiment. This effect is not directly relevant to the focus of our study, and indeed, it did not show any differences when comparing the other conditions of interest, known vs. unknown and match vs. mismatch.
