# Supplementary material for: Words before pictures: the role of language in biasing visual attention
Source: Front Psychol. 2024 Dec 18;15:1439397. doi: 10.3389/fpsyg.2024.1439397 (PMC11688633; doi:10.3389/fpsyg.2024.1439397)
Supplement: Supplementary file 1 [file Table_1.docx]

**Supplementary materials**

All stimuli, data and the analysis pipeline are openly available at the repository link https://osf.io/qt3bf/?view_only=cd4f8c1a5eca4ab493d0dff5d8e7d49e.
